# Immunohistochemical evidence for an endocrine/paracrine role for ghrelin in the reproductive tissues of sheep

**DOI:** 10.1186/1477-7827-3-60

**Published:** 2005-10-31

**Authors:** David W Miller, Joanne L Harrison, Yvonne A Brown, Una Doyle, Alanna Lindsay, Clare L Adam, Richard G Lea

**Affiliations:** 1 School of Veterinary and Biomedical Sciences, Murdoch University, South Street, Murdoch, WA, Australia; 2 Sustainable Livestock Systems Group, Scottish Agricultural College, Bucksburn, Aberdeen, UK; 3 School of Biological Sciences, University of Aberdeen, Aberdeen, UK; 4 Early Life Nutrition Group, Rowett Research Institute, Greenburn Rd, Bucksburn, Aberdeen, UK

## Abstract

**Background:**

The gut hormone, ghrelin, is involved in the neuroendocrine and metabolic responses to hunger. In monogastric species, circulating ghrelin levels show clear meal-related and body weight-related changes. The pattern of secretion and its role in ruminant species is less clear. Ghrelin acts via growth hormone secretagogue receptors (GHSR-1a) to alter food intake, fat utilization, and cellular proliferation. There is also evidence that ghrelin is involved in reproductive function. In the present study we used immunohistochemistry to investigate the presence of ghrelin and GHSR-1a in sheep reproductive tissues. In addition, we examined whether ghrelin and GHSR-1a protein expression is developmentally regulated in the adult and fetal ovine testis, and whether there is an association with markers of cellular proliferation, i.e. stem cell factor (SCF) and proliferating cell nuclear antigen (PCNA).

**Methods:**

Antibodies raised against ghrelin and its functional receptor, GHSR-type 1a, were used in standard immunohistochemical protocols on various reproductive tissues collected from adult and fetal sheep. GHSR-1a mRNA presence was also confirmed by in situ hybridisation. SCF and PCNA immunoexpression was investigated in fetal testicular samples. Adult and fetal testicular immunostaining for ghrelin, GHSR-1a, SCF and PCNA was analysed using computer-aided image analysis. Image analysis data were subjected to one-way ANOVA, with differences in immunostaining between time-points determined by Fisher's least significant difference.

**Results:**

In adult sheep tissue, ghrelin and GHSR-1a immunostaining was detected in the stomach (abomasum), anterior pituitary gland, testis, ovary, and hypothalamic and hindbrain regions of the brain. In the adult testis, there was a significant effect of season (photoperiod) on the level of immunostaining for ghrelin (p < 0.01) and GHSR-1a (p < 0.05). In the fetal sheep testis, there was a significant effect of gestational age on the level of immunostaining for ghrelin (p < 0.001), GHSR-1a (p < 0.05), SCF (p < 0.05) and PCNA (p < 0.01).

**Conclusion:**

Evidence is presented for the presence of ghrelin and its receptor in various reproductive tissues of the adult and fetal sheep. In addition, the data indicate that testicular expression of ghrelin and its receptor is physiologically regulated in the adult and developmentally regulated in the fetus. Therefore, the ghrelin ligand/receptor system may have a role (endocrine and/or paracrine) in the development (cellular proliferation) and function of the reproductive axis of the sheep.

## Background

Ghrelin is an acylated polypeptide hormone secreted predominantly by endocrine cells of the stomach [1,2]. Several lines of evidence implicate ghrelin in the regulation of growth hormone (GH) release, energy balance, food intake and body weight [3-6], with the effects mediated via a 7-transmembrane G protein-coupled receptor, the GH secretagogue receptor (GHSR) type 1a [2]. Evidence is also accumulating to suggest that ghrelin may play a role in the central regulation of reproduction. For example, exogenous ghrelin has been shown to inhibit luteinising hormone (LH) secretion in rats, both *in vivo *and *in vitro *[7,8], and immunoreactivity and gene expression for ghrelin and its functional receptor have been found in the hypothalamic region of the rat and human brain, an area known to be important in the control of reproduction [2,9-11]. There may also be peripheral (paracrine) effects of ghrelin on the reproductive axis, with reports of the ghrelin ligand/receptor system being present in rat and human gonads [12-15]. Although ruminant species also appear to utilise the ghrelin system to modulate neuroendocrine and metabolic responses to hunger [1,6,16-20], little is known of the tissue distribution of ghrelin and its receptor, nor of its link to the reproductive axis in these species.

Although ghrelin is predominantly produced by the stomach, gene expression for ghrelin and its receptor in human and rat tissues indicates a widespread distribution in various organs, including the intestine, heart, kidney, liver, lung, pancreas, placenta, pituitary, gonads and brain [21-23]. The peripheral role of ghrelin is unclear, although it has been indicated that it is involved in the control of cellular apoptosis and proliferation [24-29]. The expression of ghrelin receptor in the rat testis has also been shown to be up-regulated around the time of pubertal gonad development [30]. In sheep, the non-breeding season (which is primarily regulated by photoperiod) is associated with a marked reduction in testis weight, fewer germ cells maturing beyond the spermatocyte stages, and decreased steroidogenic activity of the Leydig cells [31]. The possible paracrine role of ghrelin in the testis in this seasonal process has not been investigated.

Development of the reproductive organs during fetal and post-natal life is essential for normal sexual function in adulthood. In rats, it is known that the fetus and placenta secrete ghrelin, and that the ghrelin receptor is present in fetal tissues [32-35]. It is speculated that alteration in maternal, placental or fetal ghrelin during pregnancy, such as that caused by maternal feed restriction [36-38], might contribute to programming of adult infertility via central or peripheral mechanisms. One such mechanism could involve a link between ghrelin and cell proliferation in the control of fetal testicular development. Ghrelin has been shown to specifically inhibit the proliferative activity of immature Leydig cells by down-regulating stem cell factor (SCF) gene expression [39]. It remains equivocal whether the ghrelin system is developmentally regulated in fetal reproductive organs, and whether there are interactions with other paracrine regulators of gonadal development such as SCF.

In the present study we used immunohistochemistry to test the hypothesis that ghrelin and its functional receptor, GHSR-1a, are present in tissues of the ovine reproductive axis, namely in adult hypothalamus, pituitary and gonads. In addition, we examined whether (a) ghrelin and GHSR-1a protein expression is developmentally or physiologically regulated in the adult and fetal ovine testis and (b) ghrelin expression is linked to proliferative activity in the developing fetal testis.

## Methods

### Animals and tissue collection

All experimental procedures involving animals were conducted under the authority of the Animals (Scientific Procedures) Act, UK, 1986 after Home Office and local ethical committee approval. Tissue samples including stomach (abomasum; i.e. control tissue), brain (hypothalamus & hindbrain), pituitary gland, testis and ovary from adult Suffolk-cross sheep collected from a local abattoir, were immersion-fixed in Bouin's solution for 6 h. Testicular tissue samples from 12 reproductively active (short-day photoperiod, 8 h light:16 h dark) and 10 reproductively regressed (long-day photoperiod, 16 h light:8 h dark) adult Soay rams (2 years old) housed under these artificial lighting conditions for 12 weeks were also collected [40] and immersion-fixed in Bouin's solution for 6 h. Testicular tissue samples for in situ hybridisation analysis of GHSR-1a mRNA expression were also collected, immediately frozen on dry ice and stored at -80C. Fetal testicular tissue samples were collected from Greyface ewes killed, as part of another study [41], on days 30, 40, 50, 70, 100 and 140 of gestation (term ≈ day 145). Fetuses (n = 7 male fetuses at each gestational age) were quickly recovered, immediately exsanguinated, and either the hind torsos were fixed intact (days 30 and 40 of gestation) or testes were fixed following dissection (on days 50, 70, 100 and 140 of gestation). Fetal testicular tissue samples were immersion-fixed in Bouin's solution for 6 h. After fixation, all tissue samples were rinsed and then stored in 70% alcohol before processing and embedding into paraffin wax using standard procedures. Sections (5 μm) were cut and mounted on poly-L-lysine coated glass slides and dried overnight at 42°C prior to immunohistochemical analysis.

### Immunohistochemistry

Tissue sections were dewaxed in Histoclear, re-hydrated through a graded ethanol series (100%, 95% &, 70%) and washed in Tris-buffered saline for 2 × 5 min. Where antigen retrieval procedures were necessary (see below), this was achieved by microwaving (800 W) the tissue sections in 0.01 M citrate buffer on full power for 3 × 5 minutes, followed by a 20 minute rest period. Sections were placed in a DAKO autostainer and incubated at room temperature with the appropriate primary antibodies as follows: (a) anti-human ghrelin (Phoenix Europe GmbH, Karlsruhe, Germany) at a 1:600 dilution for 30 mins, (b) anti-human growth hormone secretagogue receptor-1a (Phoenix Europe GmbH, Karlsruhe, Germany) at a 1:400 dilution, (c) anti-ovine SCF (kindly supplied by Dr K McNatty, Wallaceville Animal Research Station, Upper Hutt, New Zealand, and characterised by Tisdall et al. [42]), at a 1:450 dilution and (d) anti-rat PCNA (Novacastra Laboratories Ltd., Newcastle, UK) at a 1:100 dilution. Ghrelin peptide sequences are highly conserved between species [43,44] and the cross-reactivity and specificity of the anti-human ghrelin and GHSR-1a antibodies were tested using control ovine stomach tissue.

Ghrelin and SCF immunoreactivity required antigen retrieval and used the DAKO ChemMate peroxidase/DAB detection system (DakoCytomation Ltd, Ely, UK). In brief, this comprised a peroxidase block step with 3% hydrogen peroxide for 5 mins, followed by a 30 minute incubation with secondary biotinylated link antibody (ChemMate A solution) and a 30 minute incubation with peroxidase substrate (ChemMate B solution). In between each of these steps the slides were rinsed with Tris-buffered saline (TBS). Diaminobenzidine tetrahydrochloride (DAB: DakoCytomation Ltd, Ely, UK) was then applied in two 5 minute incubations. Sections were counterstained with haematoxylin Z (Vector Laboratories Ltd, Peterborough, UK). The negative controls were produced by substituting the primary antibody with normal rabbit serum at the same dilution as the primary antibody.

Ghrelin receptor (GHSR-1a) and PCNA immunoreactivity were detected using the Vectastain Elite ABC kit (Vector Laboratories Ltd, Peterborough, UK) The protocol was as described above with exception of the following: the secondary antibody was a biotinylated rabbit anti-goat IgG (Vector Laboratories Ltd, Peterborough, UK) in a 1:200 dilution. The tissue sections were then incubated with the Vectastain Elite ABC reagent (Vector Laboratories Ltd, Peterborough, UK) for 30 minutes before undergoing the final steps (as above). Negative controls were produced by substituting the primary antibody with normal rabbit or horse serum (1:200).

To determine antibody specificity, all primary antibodies were incubated overnight with immunising peptide. Following incubation, antibody-antigen preparations were centrifuged and the supernatant applied to selected tissue sections.

### Testicular GHSR-1a gene expression

Messenger RNA levels were quantified by *in situ *hybridisation using techniques previously described in detail by Mercer et al. [45]. A riboprobe complementary to GHSR-1a was generated from cloned cDNA from the hypothalamus of rat [46]. The sequence of this cDNA fragment used by Tups and colleagues [46] was compared to the same region of the ovine gene using the Multalin multiple sequence alignment website. Across this region of the GHSR-1a gene, there was 90% homology between rat and sheep.

Frozen adult sheep testicular sections (20 μm) were cut onto glass slides double-coated with gelatin and poly-L-lysine, with six or seven sections mounted on each slide. Briefly, slides were fixed in cold NBF, acetylated, and hybridised overnight at 58°C using [^35^S]-labelled cRNA probes (2 × 10^7 ^c.p.m./ml). Slides were treated with RNase A, desalted, with a final high stringency wash (30 min) in 0.1 × SSC at 60°C, dried and exposed for 4 weeks to Kodak Biomax MR Film (Kodak, Rochester, NY, USA). Autoradiographic images were captured using the Image-Pro Plus system (Media Cybernetics, Maryland, USA).

### Image and statistical analysis

Immunostained area for ghrelin and GHSR-1a in adult testis tissue from the Soay rams, and ghrelin, GHSR-1a, SCF and PCNA in fetal testis tissue was analysed using computer aided image analysis. The system was composed of a Zeiss axioplan microscope (Zeiss, West Germany) and HV-C20 Hitachi camera (Hitachi, Japan) connected to a computer running Image-Pro Plus system. Four sections per testis were quantified for tissue section area stained (brown colour) over six randomly selected fields of view previously shown to be sufficient to stabilise the mean and standard error [47]. The total area of positively stained cells (brown) was selected and expressed as a sum of pixels. All nuclei were then selected (brown = immunopositive + blue = haematoxylin Z) and the positively stained cells were expressed as a percentage of the total.

Image analysis data were subjected to one-way ANOVA, with differences between testicular immunostaining levels of ghrelin, GHSR-1a, SCF and PCNA at different stages of gestation in the fetuses determined by Fisher's least significant difference.

## Results

### Antibody specificity

**Figure 1:**
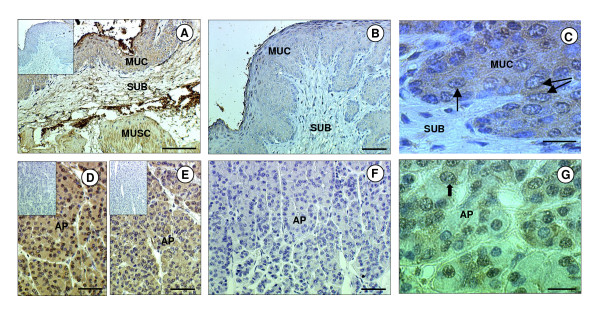
**Immunohistochemical localisation of ghrelin and GHSR-1a in the sheep stomach and pituitary gland**. Photomicrographs of sheep stomach (abomasum) and anterior pituitary (AP) sections immunostained with antibodies against either ghrelin or GHSR-1a. (A) Stomach section showing positive staining for ghrelin (brown) in tunica mucosa (MUC), tunica submucosa (SUB) and tunica muscularis (MUSC). (B) Positive immunostaining in the stomach was abolished when the antiserum was pre-incubated with the immunising peptide (ghrelin). (C) High magnification micrograph of MUC showing the cytoplasmic and perinuclear (arrow) nature of the immunostaining for ghrelin. (D) AP section showing positive staining for ghrelin in most cells. (E) AP section showing positive staining for GHSR-1a in most cells. (F) Positive immunostaining for GHSR-1a in the AP was abolished when the antiserum was pre-incubated with the immunising peptide. (G) High magnification micrograph of the AP showing the cytoplasmic and perinuclear (arrows) nature of the immunostaining for GHSR-1a. The scale bar of A represents 100 μm, for B, D, E, F they represent 50 μm, and for C, G they represent 20 μm. The insert is the negative control.

The specificity of the three antibodies (anti-ghrelin, anti-GHSR1a and anti-SCF) was confirmed by the absence of immunostaining when the primary antibody was replaced with serum from the species in which the antibody was raised. Additionally, immunostaining was abolished when the antisera were pre-incubated with the immunising peptide (Fig. 1). In all tissues investigated, the ghrelin immunopositive cells exhibited perinuclear staining and/or more widespread cytoplasmic staining. At high magnification it was confirmed that the nuclei were indeed negative (Fig. 1). The GHSR-1a and SCF positive cells both exhibited cytoplasmic staining.

### Ghrelin and GHSR-1a in adult sheep tissues

**Figure 2:**
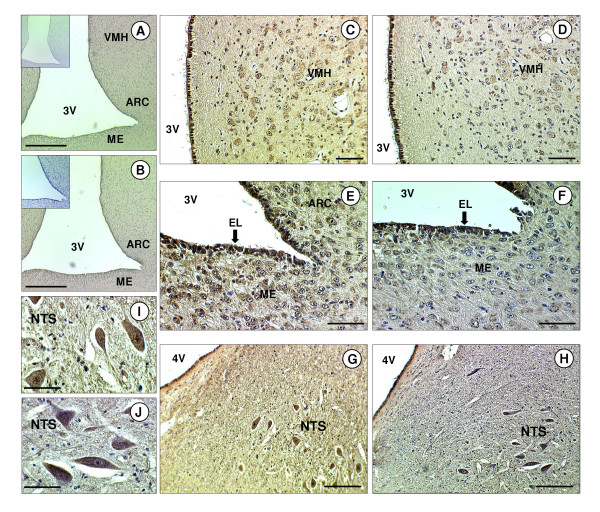
**Immunohistochemical of ghrelin and GHSR-1a in the sheep brain**. Photomicrographs of sheep brain sections. (A) Hypothalamic section including the third ventricle (3V) showing positive staining for ghrelin in the median eminence (ME), arcuate nucleus (ARC) and ventromedial hypothalamus (VMH). (B) Hypothalamic section showing positive staining for GHSR-1a in the ME, ARC and VMH. (C) High magnification micrograph showing the staining for ghrelin in the VMH. (D) High magnification micrograph showing the staining for GHSR-1a in the VMH. (E) High magnification micrograph showing the staining for ghrelin in the ARC, ME and ependymal lining (EL) of the 3V. (F) High magnification micrograph showing the staining for GHSR-1a in the ARC, ME and EL. (G) Hindbrain section including the fourth ventricle (4V) showing positive staining for ghrelin in the nucleus of the tractus solitarus (NTS). (H) Hindbrain section showing positive staining for GHSR-1a in the NTS. (I) High magnification micrograph showing the staining for ghrelin in the NTS. (J) High magnification micrograph showing the staining for GHSR-1a in the NTS. The scale bars of A, B represent 150 μm, for C, D, E, F G, H they represent 50 μm, and for I, J they represent 20 μm. The insert is the negative control.

**Figure 3:**
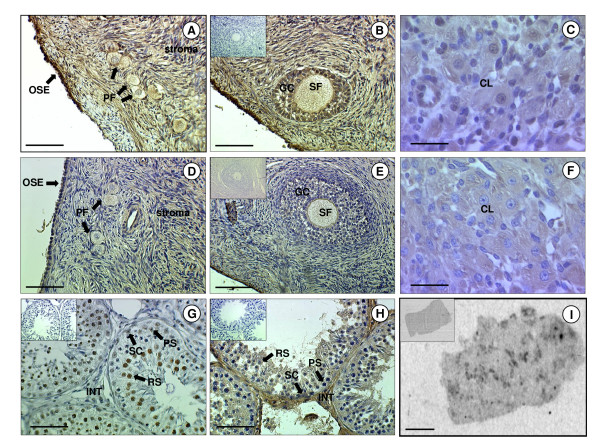
**Immunohistochemical and *in situ *localisation of ghrelin and GHSR-1a in sheep gonads**. Photomicrographs of sheep ovarian and testicular sections. (A) Ovarian section showing positive immunostaining for ghrelin in the stroma, primordial follicles (PF) and the ovarian surface epithelium (OSE). (B) Ovarian section showing positive immunostaining for ghrelin in secondary follicles (SF) and granulosa cells (GC). (C) High magnification micrograph showing positive immunostaining for ghrelin in the corpus luteum (CL). (D) Ovarian section showing positive immunostaining for GHSR-1a in the stroma, PF and OSE. (E) Ovarian section showing positive immunostaining for GHSR-1a in the SF and GC. (F) High magnification micrograph showing positive immunostaining for GHSR-1a in the CL. (G) Testicular section of seminiferous tubules showing positive immunostaining for ghrelin in the Sertoli cells (SC), pre-spermatogonia (PS), round spermatids (RS) and in the interstitium (INT). (H) Testicular section of seminiferous tubules showing positive immunostaining for GHSR-1a in the SC, PS, RS and INT. (I) Autoradiograph of adult testis sections following *in situ *hybridisation to an antisense ^35^S-labelled riboprobe to GHSR-1a mRNA showing hybridisation mainly in the interstitial areas between the seminiferous tubules. The scale bars of A, B, D, E, I represent 50 μm, and for C, F, G, H they represent 150 μm The insert is the negative control (*in situ *sense control for I).

**Figure 4:**
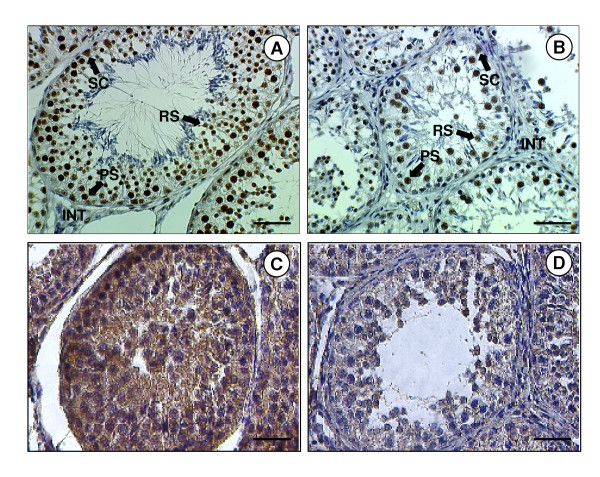
**Immunolocalisation of ghrelin and GHSR-1a in the testes from adult sheep maintained in either short- or long-day photoperiods**. Photomicrographs of adult sheep testicular sections kept for 12 weeks in either a short day (SD) or long day photoperiod (LD). (A) Section from a SD sheep showing positive staining for ghrelin in the interstitium (INT), Sertoli cells (SC), pre-spermatogonia (PS) and round spermatids (RS). (B) Section from a LD sheep showing reduced staining for ghrelin in the testis compared to the SD sheep. (C) Section from a SD sheep showing positive staining for GHSR-1a. (D) Section from a LD sheep showing reduced staining for GHSR-1a in the testis compared to the SD sheep. (E) The scale bars of A, B, D, E represent 50 μm.

There was general immunopositive staining for ghrelin and GHSR-1a throughout hypothalamic region of the brain, including the median eminence (ME), arcuate nucleus (ARC), ventromedial hypothalamus (VMH) and ependymal lining (EL) of the third cerebral ventricle (Fig. 2). Ghrelin and GHSR-1a were also present in the hindbrain where they were found in distinct neuronal bodies in the nucleus tract solitarus (NTS). The immunoreactivity of ghrelin in the NTS neuronal bodies showed general cytoplasmic staining with some nuclear/perinuclear staining also detectable. (Fig. 2). In the anterior pituitary gland, ghrelin and GHSR-1a immunoreactivity was also present, with GHSR-1a showing cytoplasmic and perinuclear staining in some cells (Fig. 1) In the adult ovary, ghrelin and GHSR-1a were immuno-localised to ovarian follicles at all developmental stages (primordial, primary, secondary, pre-antral and antral). Both proteins were also co-localised to the granulosa cells and some staining was observed in the thecal cells of the larger follicles (Fig. 3). Ghrelin and GHSR-1a immunostaining was also present in the luteal cells of the corpus luteum. In some of the sections their appeared to be some positive staining for ghrelin and GHSR-1a on the oocyte (especially in the larger oocytes). However, this finding was not consistent across all oocytes. In the adult testis, ghrelin immunostaining was predominant in the germ and Sertoli cells, with the germ cells showing intense perinuclear staining (Fig 3). Moreover, there appeared to be more intense ghrelin staining in the germ cells in all developmental stages prior to the first meiotic division. Lower level immunostaining was also observed in the interstitial tissue (where Leydig cells are localised: Fig 3g). GHSR-1a protein was also detected in cord Sertoli, and germ cells. (Fig 3h, Fig 4c,d). In contrast to ghrelin, staining for the receptor appeared to be more predominant in the interstitium (Fig 3h: GHSR-1a v Fig 3g: ghrelin). In addition, in-situ hybridisation for GHSR-1a mRNA showed clear rings of hybridisation within the testis, indicative of gene expression in the interstitial tissue around the seminiferous tubules (Fig. 3i).

### Effect of photoperiod on Ghrelin and GHSR-1a in the adult Soay testis

**Figure 5:**
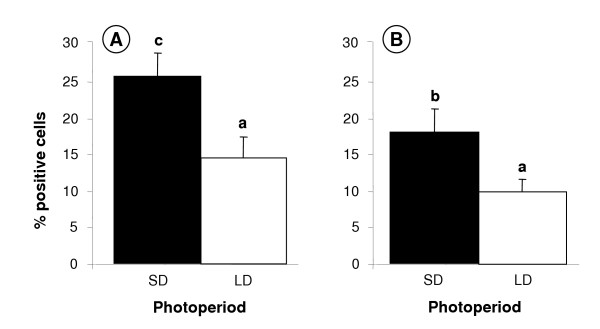
**Effect of photoperiod on the percentage of cells positively immunostained for ghrelin and GHSR-1a in adult sheep testes**. Ghrelin (A) and GHSR-1a (B) in adult sheep testes (short days, SD versus long days, LD). Values with different alphabetical superscripts are significantly different to one another: a versus b = p < 0.05; a versus c = p < 0.01. Values are means ± S.E.M.

Testis size was reduced in the LD group (data not shown), as expected [40]. Ghrelin and GHSR-1a immunostaining was demonstrated in germ, Sertoli and interstitial cells of adult Soay testes collected in both the short-day (SD: reproductively active) and long-day (LD: reproductively regressed) photoperiods (Fig. 4). Comparison between the two groups indicated that the number of ghrelin and ghrelin receptor positive cells was reduced in the testes from the LD (reproductively regressed) Soay rams. This was verified after statistical analysis of the image analysis data indicating a significant increase in immunostaining levels in the SD photoperiod for both ghrelin (p < 0.01) and GHSR-1a (p < 0.05) (Fig. 5). More detailed histological examination indicated that the decreased ghrelin staining was predominantly associated with reduced staining of the testicular cords (Sertoli and germ cells). In contrast, photoperiod appeared to influence GHSR-1a staining in both the cords and interstitial areas.

### Ghrelin, GHSR-1a, SCF and PCNA in the fetal sheep testis

**Figure 6:**
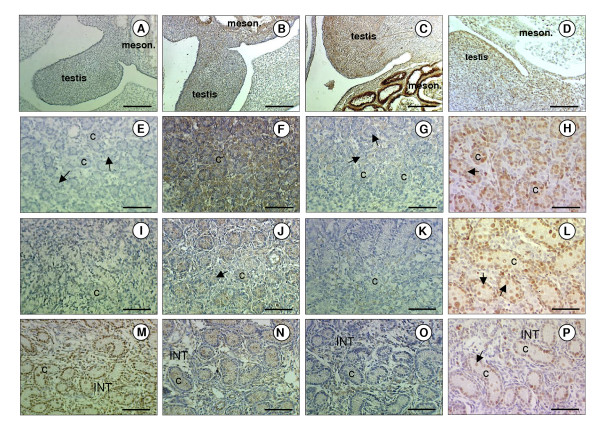
**Immunolocalisation of ghrelin and GHSR-1a in fetal sheep testes**. Photomicrographs of fetal sheep testicular sections. (A, B, C, D) Sections from fetuses at day 30 of gestation showing positive immunostaining for ghrelin (A), GHSR-1a (B), SCF (C) and PCNA (D) in the fetal testis and mesonephros (meson.). (E, F, G, H) Testicular sections from fetuses at day 50 of gestation showing positive immunostaining for ghrelin (E), GHSR-1a (F), SCF (G) and PCNA (H). (I, J, K, L) Testicular sections from fetuses at day 70 of gestation showing positive immunostaining for ghrelin (I), GHSR-1a (J), SCF (K) and PCNA (L). (M, N, O, P) Testicular sections from fetuses at day 140 of gestation showing positive immunostaining for ghrelin (M), GHSR-1a (N), SCF (O) and PCNA (P) in the seminiferous cords (C) and interstitium (INT). Arrows depict germ cells. The scale bars of A, B, C, D represent 150 μm, and for the rest they represent 100 μm.

The ovine fetal testis is visible as an outgrowth of undifferentiated cells from the mesonephros at gestational day 30 (Fig. 6a–d). At this stage it was impossible to visually differentiate between somatic and germ cells. At day 50 (Fig. 6e–h), small testicular cords were distinguishable towards the periphery of the gonadal tissue, and germ cells were present surrounded by cells that will constitute the Sertoli cell population at a later gestational stage. At day 70 (Fig. 6i–l) the testicular cords were more pronounced. Germ and Sertoli cells were identifiable within the cords. Interstitial cells, probably Leydig cells and their precursors, were clearly identifiable.

At day 30, low level immunostaining for ghrelin was observed in the gonadal/mesonephros complex (Fig. 6a). From day 70 (Fig. 6i) onwards ghrelin staining intensity increased, and at day 140 (Fig. 6m), ghrelin was clearly localised to the Sertoli, germ and some interstitial cells. Maximum GHSR-1a immunostaining intensity was evident at day 50 (Fig. 6b), although lower levels were present at all other gestational time points. From day 70 onwards, positive staining for GHSR-1a was restricted to the Sertoli and interstitial cells (Fig. 6j,n)

Cells stained for SCF were most prominent in the fetal testis at day 30 (Fig. 6c). The later gestational stages appeared to have fewer cells of lower staining intensity. From day 70 onwards SCF immunopositive cells were localised to the interstitium and to the endothelial cells of the blood vessels. PCNA-immunopositive cells were detected in the gonadal/mesonephros complex at day 30 (Fig. 6d) and was predominantly localised to the seminiferous cords at the later gestational ages examined. Positive PCNA staining was seen in the Sertoli and germ cells and staining was also evident in the Leydig cell containing interstitial area (Fig. 6h,l,p).

**Figure 7:**
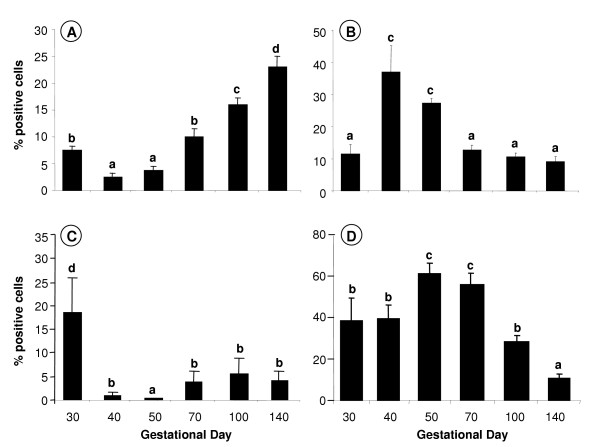
**Effect of gestational age on the percentage of fetal testicular cells positively immunostained for ghrelin, GHSR-1a, SCF and PCNA**. Ghrelin (A), GHSR-1a (B), SCF (C) and PCNA (D) in fetal sheep testes (days 30 – 140 of gestation; n = 7 at each gestational age). Values with different alphabetical superscripts are significantly different to one another: a versus b, b versus c, and c versus d = p < 0.05; a versus c, and b versus d = p < 0.01; a versus d = p < 0.005. Values are means ± S.E.M.

There was a significant overall effect of gestational age on the immunostaining levels of ghrelin in the fetal testis (Fig. 7a: p < 0.001), GHSR-1a (Fig. 7b: p < 0.05), SCF (Fig. 7c: p < 0.05) and PCNA (Fig. 7d: p < 0.01). At day 30, ghrelin staining levels were about 2 times higher (p < 0.05) than the nadir levels at days 40 and 50. From days 70 to 140, ghrelin staining levels increased reaching peak fetal levels at day 140 that were about 7 times higher than the nadir level at day 40 (p < 0.005). In comparison, GHSR-1a immunostaining intensity levels appeared to have the opposite temporal pattern to ghrelin, with the peak levels at days 40 to 50, which were about 3 to 4 times higher than at any other time during gestation (p < 0.05). The fetal staining intensity pattern for SCF was similar to ghrelin, with immunostaining intensity levels at day 30 being 3 to 4 times higher than the nadir levels at day 40 to 50 of gestation (p < 0.05), whilst the staining intensity pattern for PCNA was similar to GHSR-1a, with immunostaining intensity levels peaking at day 50 to 70, which were about 50 to 80% higher than at days 30, 40 and 100 (p < 0.05), and about 4 times higher than at day 140 (p < 0.01).

## Discussion

The present study provides immunohistochemical evidence for the presence of ghrelin and its functional receptor, growth hormone secretagogue receptor (GHSR-1a), in reproductive tissues of the sheep. Novel data are presented that indicate that ghrelin and its functional receptor, GHSR-1a, are regulated during development of the ovine fetal testis and by seasonal developmental changes in the adult testis. Moreover, the developmental pattern of expression corresponds with the postulate by Barreiro and colleagues [39] that the ghrelin system is linked to the proliferative activity of germ and somatic cells in the testis.

In the sheep brain, ghrelin and GHSR-1a immunoreactivity were demonstrated in the hypothalamic region, including the median eminence (ME), arcuate nucleus (ARC), ventromedial hypothalamus (VMH) and ependymal lining (EL) of the third cerebral ventricle. The presence of ghrelin and GHSR-1a in the hypothalamus is consistent with its role in the regulation of food intake [48]. However, the hypothalamic localisation also corresponds with ghrelin's putative role in the control of LH secretion, as neurons that secrete gonadotrophin-releasing hormone (GnRH) are also located in these hypothalamic regions [49,50]. Central administration of ghrelin has been shown to rapidly suppress pulsatile LH secretion in ovariectomized rats [8]. Ghrelin and GHSR-1a mRNA and protein [2,9-11,51-53] have previously been found in the rat and human hypothalamus. In the sheep hindbrain, ghrelin and GHSR-1a immunostaining was also identified in neuronal cell bodies within the nucleus tract solitarus (NTS), but not in the area postrema. Similarly, an immunohistochemical study in rats by Lin et al. [54] revealed that the GHSR-1a was expressed in the neuronal cells of the NTS and the dorsal motor nucleus of the vagus, but not in the cells of the area postrema. Using c-fos immunohistochemistry, Lawrence et al. [55] demonstrated that central ghrelin administration activated two regions of the brainstem, the NTS and the area postrema. It is tempting to speculate that ghrelin may affect food intake and the neuroendocrine system at the level of the NTS, a central nervous system (CNS) site that receives primary vagal afferent input from the digestive tract and acts as a neuronal relay station to the hypothalamus [56]. However, it could also be possible that ghrelin is simply participating in the central regulation of gastric acid secretion via the vagus system. ICV administration of ghrelin in rats increases gastric acid output in a dose-dependent manner, and vagotomy and the administration of atropine abolishes gastric acid secretion induced by ghrelin [57].

In the sheep anterior pituitary gland, ghrelin and GHSR-1a were ubiquitously expressed. Ghrelin gene expression has been found in the pituitary glands of rodents, pigs and humans [13,21,58]. It is evident from the literature that the main action of ghrelin at the level of the pituitary is the release of GH [59,60]. Indeed, GH secretagogue receptors were first identified in the pituitary by the ability of enkephalin analogues to stimulate GH release [60]. However, ghrelin may also be involved in somatotroph cell differentiation since ghrelin regulates pituitary-specific transcription factor (Pit-1) expression in the rat pituitary [62]. Somatotroph cell-specific expression of the GH gene is dependent on Pit-1 [63,64]. Ghrelin has also been shown to exert a proliferative effect on a rat pituitary somatotroph cell line via the mitogen-activated protein kinase (MAPK) pathway [27]. Interestingly, ghrelin may also play a role in LH secretion at the level of the pituitary as it has been shown that ghrelin stimulates gonadotrophin release from rat pituitary cells *in vitro *[7]. Particularly evident in the sheep pituitary tissue was the finding that GHSR-1a immunoreactivity in some cells of the pituitary showed intense perinuclear staining. This staining pattern may arise if the polyclonal antibody is detecting the ligand/receptor complex and is consistent with the finding of Camina et al. [65] that the ghrelin/GHSR-1a complex progressively disappears from the plasma membrane after binding of the ligand and accumulates in the perinuclear region.

Ghrelin and GHSR-1a immunostaining were detected in sheep ovarian follicles at all developmental stages, mainly in the granulosa cells. Caminos et al. [13] and Gaytan et al. [14] also found immunoreactivity for ghrelin and its receptor in the rat and human ovary, though only weak ghrelin staining was evident in the follicles. Strong ghrelin and GHSR-1a immunostaining was evident in corpora lutea (CL) of the sheep ovary, similar to the findings in the rat and human CL. Caminos et al. [13] found dynamic changes in the profile of ghrelin expression during the oestrous cycle and throughout pregnancy in rats, suggesting a precise regulation of ovarian expression of ghrelin. Gaytan and colleagues [14] have suggested a potential, yet unproven relationship between GHSR-1a expression and follicle growth, since expression of the receptor in somatic cells derived from ovarian follicles roughly parallels follicular development in the human ovary. There was additional evidence for ghrelin and GHSR-1a peptide expression within the ovarian surface epithelium (OSE) in the present study. Gaytan et al. [66] have also recently shown GHSR-1a peptide expression in human OSE. During ovulatory cycles the OSE is subject to a series of injury and repair processes associated with follicular rupture and CL formation, which involve natural inflammatory events. Pro-inflammatory IL-1α produces an increase in mRNA levels of 11 betahydroxysteriod dehydrogenase type 1 (11βHSD1) in OSE cells, encoding the steroid dehydrogenase that reversibly reduces cortisone to anti-inflammatory cortisol [67]. Tan and colleagues [68] have recently demonstrated the ability of ghrelin to stimulate 11βHSD1 in the human ovary, providing evidence to support the theory that ghrelin plays an immunomodulatory role in OSE cells of the ovary via an anti-inflammatory action.

In the adult sheep testis, strong ghrelin immunostaining was evident in the interstitial area where Leydig cells are localised. Staining was also present in the germ and Sertoli cells, with an indication of increased ghrelin immunoreactivity in the germ cells during the mitotic phases and meiotic pro-phases of the spermatogenic cycle. GHSR-1a protein was detected in the interstitial Leydig cell containing area of the testis, as well as in the Sertoli and germ cells within the tubules, and the pattern of GHSR-1a mRNA expression across the testis indicated that the mRNA was present in the interstitial area and around the periphery of the tubules. Ghrelin immunostaining has been demonstrated in interstitial Leydig cells and, at lower intensity, in Sertoli cells of the rat [15]. Ghrelin and its receptor are present in the human testis, but in contrast to the ovine data, ghrelin protein is not detectable in germ cells at any stage of spermatogenesis [21]. There appears, therefore, to be some species differences in the localisation of ghrelin protein in the testis. Ghrelin has been shown to dose-dependently inhibit testicular testosterone secretion *in vitro*, and to modulate Leydig cell proliferation *in vivo *and the expression of relevant testicular genes, such as that encoding stem cell factor (SCF) [69]. In the testicular samples collected from Soay rams maintained in different photoperiods (short day = reproductively active, and long day = reproductively regressed), it was evident that ghrelin and GHSR-1a were up-regulated in the short-day photoperiod. This finding corresponds with the postulated role of ghrelin in the proliferation of somatic and germ cells in the testis [15,30]. It has been suggested that the expression of ghrelin peptide in mature Leydig cells in the rat testis is under the hormonal regulation of pituitary LH [12]. Whether this action is carried out directly, or is mediated by LH-driven locally produced factors, such as testosterone, requires further investigation. It is pertinent that this observation accompanies an increase in testis size which characterises reproductively active animals [40].

In the fetal sheep testis, the present findings indicate that the expression of ghrelin and GHSR-1a protein is linked to gestational age. Differentiation of the fetal gonad begins with the development of the gonadal ridges from thickening of the ventrolateral surface of the embryonic mesonephros. The genital ridge is composed of somatic cells from the mesonephros and migratory primordial germ cells originating from the extraembryonic mesoderm. In the fetal sheep, morphological sexual differentiation of the gonads begins around day 27 of gestation, and germ cell migration is complete just after day 30 [70,71]. In the present study, significant levels of ghrelin and SCF were found in the gonad/mesonephros complex at day 30 of gestation. Ghrelin has been implicated in proliferative activity in a number of tissues, including the gonad [27-29,39]. SCF is also said to be involved in proliferation, germ cell migration and survival [72]. Therefore, ghrelin and SCF may be involved in the early differentiation of the ovine fetal testis. Intratesticular injection of ghrelin has also been shown to decrease the proliferative activity of differentiating immature Leydig cells in the rat, and this response is associated with a decrease in the mRNA levels of SCF, a putative regulator of Leydig cell development [39]. In the fetal sheep, Leydig cell hyperplasia occurs between day 50 and 70 of gestation [73]. In the present study it was noted that at day 50, GHSR-1a and PCNA were significantly up-regulated at the same gestational time-point when SCF protein levels were at their lowest, i.e. at the start of Leydig cell hyperplasia. These data are consistent with the putative involvement of fetal testicular ghrelin and its functional receptor in the paracrine regulation of Leydig and germ cell development, possibly via interactions with SCF and PCNA.

## Conclusion

The present data indicate that both components (ligand and receptor) of the ghrelin signalling system are present in tissues of the reproductive axis of the sheep. The ligand and receptor are developmentally regulated in the fetal testis and physiologically regulated by photoperiod in the adult testis. These findings are consistent with both a central endocrine role and a potential peripheral paracrine/endocrine regulatory role for ghrelin in the control of reproductive tissue development and function in sheep. Further studies are needed to identify the precise functional role of the ghrelin system in the reproductive axis.

## Authors' contributions

DWM participated in the design of the studies, collection of tissues, all immunological analyses, statistical analyses and drafting the manuscript. JLH participated in the design of the studies, collection of tissues, immunological and *in situ *analyses of ghrelin and GHSR-1a, statistical analyses and drafting the manuscript. YAB participated in the immunological analysis of GHSR-1a, statistical analysis and drafting the manuscript. UD participated in the immunological analysis of SCF, statistical analysis and drafting the manuscript. AL participated in the immunological analysis of PCNA, statistical analysis and drafting the manuscript. RGL participated in the design of the studies, all immunological analyses, statistical analyses and drafting the manuscript. CLA participated in the design of the studies, collection of tissues, *in situ *analysis, statistical analyses and drafting the manuscript.
